# Identification of the Range of Nursing Skills Used to Provide Social Support for Mothers of Preterm Infants in Neonatal Intensive Care

**DOI:** 10.1155/2021/6697659

**Published:** 2021-01-07

**Authors:** Shadi Eskandari, Seyedeh Nooshaz Mirhaghjou, Maryam Maleki, Abbas Mardani, Mostafa Gholami, Celia Harding

**Affiliations:** ^1^School of Nursing and Midwifery, Guilan University of Medical Sciences, Rasht, Iran; ^2^Social Determinants of Health Research Center (SDHRC), School of Nursing and Midwifery, Guilan University of Medical Sciences, Rasht, Iran; ^3^School of Nursing and Midwifery, Shahroud University of Medical Sciences, Shahroud, Iran; ^4^Nursing Care Research Center, School of Nursing and Midwifery, Iran University of Medical Sciences, Tehran, Iran; ^5^Honorary Professor and Honorary Speech and Language Therapist,City, University of London and Royal Free Hospital NHS Foundation Trust, London, UK

## Abstract

**Background:**

Hospitalization of preterm infants in neonatal intensive care units (NICUs) is a stressful experience for parents. Iranian NICUs do not have specified levels of care, nor do they integrate supportive methods of parent support such as family-centered care approaches. This study investigated the range and types of neonatal nursing support, as perceived by mothers of preterm infants, and its association with mothers' satisfaction with infant care in the NICU.

**Methods:**

This is a descriptive, correlational study of mothers of preterm infants who were hospitalized in three different NICUs in Iran. A convenience sampling method was used. Data were collected using three questionnaires that identified (i) demographic information; (ii) social support available; and (iii) parent satisfaction with infant care.

**Results:**

Mothers (*N* = 110) generally rated the support from nurses as being moderate. Correlation analysis identified a moderate association of neonatal nurse social support domains for affirmational (*r* = 0.44) and concrete aid (*r* = 0.41), a moderately strong association for affectional support (*r* = 0.64), and total social support (*r* = 0.60) with mothers' satisfaction.

**Conclusion:**

There were positive associations between social support from nurses and mothers' satisfaction with the care of their infants. Therefore, planning to promote and create opportunities for neonatal nurses to support mothers in NICU is important to promote increased maternal satisfaction in infant care.

## 1. Introduction

Infant birth is challenging for all parents, especially mothers [1]. Specifically, emotions and stress levels of parents can increase in severity when an infant is born prematurely or has health problems that require admission to a neonatal intensive care unit (NICU) [2]. Mothers are considered the main source of care for their infants [3]. However, following preterm birth or postbirth health problems requiring NICU admission, mothers may find themselves in crisis [4, 5]. Coming to terms with a fragile preterm infant, alongside the loss of the expected role, contributes to parental feelings of loss and stress [6, 7]. This experience can also cause parents to experience physical and psychological problems such as sleeplessness, depression, despair, and emotional turmoil [2, 5, 7].

Previous research has highlighted that maternal stress in this situation can be increased further when social support is lacking [2]. In order to cope, parents need to receive social support from neonatal staff [4]. The Cockcroft study identified the importance of the following: involving families in the care of their infants, ensuring inclusive practice skills are implemented by staff, and providing social support [8]. Social support is a multidimensional concept which includes categories of affection support, affirmation support, and concrete aid [9, 10].

Nurses play an important role in supporting parents and helping them cope with their child's illness both during hospitalization and upon discharge [11]. By encouraging parental involvement in infant care, giving honest answers to questions, actively listening to parent concerns and questions, and discussing fears and expectations of the hospitalization, parents are enabled to cope and to learn to care for their infant [4, 12]. Parents value the core nursing behaviours of social support, empathy, empowerment, encouragement to link with other NICU parents, psychological help, and education to help understand the complexities of their child's needs [13, 14].

Learning to be a parent of a preterm infant in a neonatal unit requires social support, which includes the accurate relaying of information, inclusion in decision-making, learning to care directly for the infant, and a therapeutic relationship with the nursing staff to minimize stress and anxiety [15]. An important task for neonatal nurses is to create a supportive environment by respecting parents' views and needs, answering parent questions honestly, by encouraging parents while skills are being learned in a state of vulnerability [16]. When given support and guidance to understand complex medical information, a crucial learning environment is created for parents to progress with their infant's care [8, 9, 12]. Oommen and colleagues identified that social support for parents is particularly essential during the critical period of admission and that the nursing staff have an important role to play in highlighting and communicating to parents the importance of their infant [17]. However, parent communication with and support from nurses have been described as lacking in social support and clear information [18].

In today's society, measuring patient satisfaction has become integral to the evaluation of health care services [19]. In this regard, social support plays an important role in the satisfaction of care [20]. Tsironi and colleagues concluded that the knowledge and information that parents received from the care staff were valuable to them and that they enabled them to feel less stressed [21]. Satisfaction is reflective of a consumer's personal preferences, expectations, and realities of the care received [9]. Quality of care, from the parents' perspective, plays an important role in the development and improvement of health services' performance and image. Patient or parent satisfaction is also a significant indicator that evaluates the care quality provided by health care services [21].

Parents of NICU infants who have received clear information and who are involved in decision-making about their infants feel more in control [4]. Nevertheless, the findings of an integrative review revealed that parents with premature infants consistently reported unmet information and support needs [22]. In addition, limited access to privacy and poor communication with nursing staff can impact on building a trusting parent–nurse relationship [14]. Moreover, in a study undertaken in Iran, most parents felt that they did not have enough information about their infant [23]. Some research identified that limited access to adequate social support can have an impact on parents' abilities to cope with their preterm infant [24]. These factors can reduce parental satisfaction with the nursing care of infants in NICUs.

This study was undertaken in Iran. In Iranian NICUs, it is standard practice that only mothers are allowed to stay in the unit, and fathers are permitted to visit the baby for limited visiting times during the day [25]. Also, unlike many NICUs in Europe and the United States, Iranian units do not have specified levels of care, nor do they always integrate supportive methods of parent support such as family-centered care, despite the fact that mothers are able to be with their infant for the duration of their stay [26]. Therefore, the aim of this study was to improve knowledge of the specific types of social support provided by nurses to mothers of preterm infants and to determine its association with mothers' satisfaction with the care of their preterm infants in NICUs based in Iran.

## 2. Materials and Methods

### 2.1. Design and Setting

This descriptive, correlational study focused on mothers of preterm infants who were hospitalized in three NICUs within hospitals affiliated to a medical university in two urban areas of Iran from November 2016 to February 2017.

### 2.2. Participants and Sampling

Eligibility criteria for the recruitment of samples were as follows: mothers of infants with a gestational birth age between 28^0/7^ weeks and 36^6/7^ weeks from the mothers' last menstrual period, and those without congenital anomalies or disorders associated with prematurity such as gestational hypertension, gestational diabetes, and genetic disorders. In addition, mothers who had previously experienced a NICU stay, who had experienced the death of an infant, or who had difficulties with literacy were excluded. The required sample size was calculated to be 106 based on a similar study [20] with 95% confidence level, 90% test power, while assuming that the correlation coefficient between providing social support for mothers with premature infants and their satisfaction with the care be at least 0.31 to be statistically significant.

To recruit participants, a convenience sampling method was used. The researchers contacted the identified NICUs. Then, they approached the mothers whose infants were being prepared for discharge and provided an explanation about the study. Next, participants who met the inclusion criteria enrolled were in the study after being given 24 hours to consider participation. In total, 110 mothers were recruited to participate in the study.

### 2.3. Instruments

#### 2.3.1. Demographic Data Questionnaire

A questionnaire was designed by the researchers through a review of the literature to examine relevant demographic information about participants. The first part of the questionnaire contained information regarding infants' demographic data including gestational birth age, gender, weight at birth, birth rank, type of birth (singleton birth or multiple birth), and length of hospitalization. The second part contained questions about the infant's family including the parents' age, parents' education level, parents' employment, and maternal delivery type.

#### 2.3.2. The Social Support Questionnaire

The Social Support Questionnaire [27] was used to investigate the different types of support provided by nurses to participants (Supplementary File 1). This tool consists of 21 questions in three subscales including [affirmational support] (validating positive actions) (7 questions) [concrete aid] (specific things which provide assistance, e.g., money and time available to participate in care) (6 questions) and [affectional support] (liking, empathy, and respect) (8 questions). Questions are rated on a seven-point scale from 0 (no need for support) to 6 (a great deal of support). For each subscale and total social support, a score of 0 to 2 indicates low support, a score of 2 to 4 indicates moderate support, and a score above 4 indicates high support. It had been reported that the Farsi version of this questionnaire has acceptable validity. Also, internal consistency (Cronbach's alpha coefficient) was reported from 0.76 to 0.88 on its subscales [20]. Also, in this study, Cronbach's alpha coefficient for the questionnaire subscales ranged from 0.74 to 0.81.

#### 2.3.3. The Neonatal Instrument of Parent Satisfaction (NIPS) Questionnaire

The Neonatal Instrument of Parent Satisfaction (NIPS) questionnaire [28] was used to assess mothers' satisfaction with the care of preterm infants (Supplementary File 1). It consists of 24 questions rated by a seven-point Likert scale (from 1 to 7). According to the literature, a score of 1 to 3.49 indicates low satisfaction, a score of 3.5 to 5.24 indicates moderate satisfaction, and a score above 5.25 shows high satisfaction [20].

In order to determine the content validity of the NIPS questionnaire used in this study, the Farsi version of the NIPS questionnaire was scrutinized and completed by ten academic nurses. After collecting comments, the content validity index (CVI) and content validity ratio (CVR) were calculated. The results showed that the CVR and CVI for this questionnaire were 0.7–1 and higher than 0.9, respectively. In addition, to assess the reliability of this questionnaire, parallel-form reliability measures were used. In doing this, two forms of the NIPS questionnaire were made, which differed only in the layout of questions. Both forms were completed within one hour during an interview with the mothers whose infant was admitted to the NICU. The correlation coefficient (*r* = 0.971, (*N* = 20), *p* < 0.001) and the reliability coefficient (97.9%) were reported as suitable. Additionally, Cronbach's alpha coefficient in our samples was calculated as 0.77, indicating high internal consistency.

### 2.4. Data Collection

Sampling was done in the morning and early evening shifts (before the discharge of the infants). Questionnaires were completed through interviews with mothers of infants who had met the eligibility criteria by a researcher in a private location of the hospital. Mothers gave written consent to participate after reading and discussing the aims of the study. They were informed that they could withdraw from the study at any time. Data from mothers (*n* = 18) who did not sustain participation were removed from the results.

### 2.5. Ethical Consideration

The Social Determinants of Health Research Center of Guilan University of Medical Sciences approved the study protocol. This study had the following approval code: 922362.

### 2.6. Data Analysis

Data were analyzed using the SPSS software, version 22. For the descriptive variables, the mean and standard deviations for quantitative variables, as well as frequency and percentage for qualitative variables, were recorded. The normality of the data was tested through the Kolmogorov–Smirnov test. As data were not normally distributed, the Friedman test was used to analyze the dimensions of social support provided by nurses. The relationships between social support from a nurse in all investigated domains and mothers' satisfaction were assessed through the use of the Spearman's–Rho rank correlation test. The significance level for all analyses was set at less than 0.05.

## 3. Results

The demographic characteristics of the participants were categorized into two groups of infant-related and family-related variables. The results highlighted that most of the preterm infants were male (57.3%), the first child in the family (66.4%), and singleton birth (76.4%). Moreover, the mean weight at birth of preterm participants was 1638.7 grams. The mean fetal age and length of hospitalization were 32.3 weeks and 25.7 days, respectively. Other demographic information is shown in Table 1.

As shown in Table 2, the majority of participants reported a moderate level of social support provided by nurses including the following subscales: affirmational (60.9%), concrete aid (61.8%), affectional (50.9%), and total social support (58.2%). The mean SD of total social support was measured as 3.78 (0.70), which indicated a moderate level of provided social support to the participants by nurses. In addition, there were no significant differences among the score of social support subscales of affirmational, concrete aid, and affectional support was seen (*p*=0.1).

According to Table 3, the majority of mothers had a moderate level of satisfaction (61.8%) followed by high satisfaction (28.2%) regarding preterm infant care received in NICUs. The mean (SD) satisfaction was 4.65 (0.83) indicating a moderate level of mothers' satisfaction with preterm infant care received.

Table 4 summarizes the relationship between nurses' social support and mothers' satisfaction with preterm infant care received in the NICUs. There was a moderate association for affirmational (rs = 0.44, (*n* = 110), *p* < 0.001) and concrete aid (rs = 0.41, (*n* = 110), *p* < 0.001) support, a moderate to strong association for affectional support (rs = 0.64, (*n* = 110), *p* < 0.001), and total social support (rs = 0.60, (*n* = 110), *p* < 0.001) with mothers' satisfaction.

## 4. Discussion

The findings of this study revealed that the majority of participants thought they received a moderate level of social support from nurses. Higher ratings of perceived social support correlated with higher maternal satisfaction ratings of preterm infant care.

The current literature highlights the importance of family-centered care approaches for families of infants receiving care in the NICU [8]. With family-centered care knowledge, neonatal nurses are best placed to be aware of and identify factors that can trigger parental stress and therefore give appropriate support [3]. In line with the findings of this study, a similar study explored mothers' perceptions of nursing support in a Jordanian context, which revealed that the level of maternal perception of nursing social support was moderate [9]. In contrast, another study showed that mothers and fathers felt they received moderate to minimal levels of social support from nurses in a postnatal ward [17]. Consistent with previous studies, our study identified that mothers received more support in the affectional domain [6, 17].

However, there was another study which found that parents of infants evaluated the support of nursing staff as high [3]. In this regard, Mousavi et al. conducted a systematic review and meta-analysis to compare the premature infant-parent support in Iran with other countries [29]. The results revealed that, in general, Iranian premature infants and parents received lower levels of support compared to other countries [29]. This lower level of social support for mothers of preterm newborns in Iranian NICUs is a significant problem which needs to be addressed.

Lower levels of social support for preterm infant mothers in Iranian NICUs can partly be explained by the shortage of nursing staff [30] as well as a more limited understanding and knowledge of the importance and need for maternal affectional support [31]. Therefore, nurses in Iran need to address and improve understanding of the importance of social support for mothers of the preterm infant in NICUs and provide holistic care, preferably within a family-centered care context.

Parental satisfaction is linked to care involving affectional support in the NICU [32]. In our study, the majority of mothers had a moderate level of satisfaction from preterm infant care in NICUs. Similarly, the results of another study conducted in Iran showed that parents' satisfaction with infant care in NICUs is at a moderate level [20]. On the other hand, many studies indicated moderately high to high levels of parent satisfaction of neonatal care in NICUs [33–35]. This diversity can be linked to the structural elements of care in Iranian hospitals when compared to other countries [36]. Family-centered care is becoming a standard model for providing care in NICUs all over the world [37]. In this approach, parents engage in their infant's care and are found to have increased satisfaction and reduced stress levels [16, 37]. However, in the Iranian health care system, there is a shortage of NICU cots for the mothers/families to stay at the bedside, additionally, there needs to be a greater focus on improving care and interventions provided within such setting [38]. There is also a shortage of skilled neonatal nursing staff [30]; a lack of awareness, knowledge, and education related to family-centered care approaches; and an overriding medicalization of infant care, which interferes with the implementation of nursing and therapeutic methods to support both infant and parent development [39]. In consequence, parental satisfaction with the care of their infants is reduced.

The results from our study also revealed that there was a strong association between social support provided by mothers and mothers' satisfaction with the care of preterm infants in the NICU. Similarly, a study found that support for parents, especially in the decision-making processes for their infant and the continuity of treatment was associated with increased satisfaction in infant care [34]. In addition, De Bernardo et al. found that parents who received support using a family-centered care model in NICUs were more satisfied with the care and experienced less stress [16]. Another study reported that nurses' support for parents was only identified in the affirmational domain (validating positive actions) as being associated with their satisfaction with infants' care [20]. This discrepancy could be explained by the fact that, unlike our study, fathers as well as mothers were included. Fathers were reported as being less likely to communicate with their infant and the nurses in NICUs; therefore, the differences are not unexpected. It seems that positive associations of nursing support and satisfaction with parent care are due to the fact that nurses advocate for their clients and can play an important role in educating and supporting mothers with infant care [10]. Weis et al. demonstrated that family-centered care can provide a structured method of delivering infant care and can also facilitate supportive communication between parents of premature infants and nurses [40]. This, in turn, improves the quality of family-centered care and the parents' ability to provide care and have positive interactions with their infants. As a result, there is increased parent satisfaction with the care of infants received in NICU and in developing confidence in learning to support and care for a preterm infant.

Our study, along with the studies discussed above, highlights that nurses have an important role in providing social support for parents, specifically for mothers in NICUs and can increase maternal satisfaction with infant care. Our study also suggests that nurses in Iranian NICUs have an opportunity to improve maternal satisfaction.

### 4.1. Limitations

To the best of our knowledge, this study is one of the few studies that simultaneously examined social support provided by nurses and mothers' satisfaction with preterm infant care in Iranian NICUs. Nevertheless, this study has limitations that should be considered and investigated in future studies. First, a convenience sampling method was used to recruit so there is a high potential for sampling bias. Secondly, using a small sample size in a specific population decreases the reliability of the findings. Lastly, caution should be exercised when generalizing findings to other countries, as there are potential cultural and environmental differences, which contribute to maternal perceptions of care.

## 5. Conclusion

Our study identified that social support provided by nurses and mothers' satisfaction with preterm neonatal care in Iranian NICUs were at a moderate level. Additionally, there was a direct relationship between social support provided by nurses in all dimensions and mothers' satisfaction with preterm infant care in Iranian NICUs. Therefore, neonatal nurses should consider providing greater attention to parent social support and family-centered care approaches in the NICU setting in order to improve parental satisfaction and provide a supportive environment for both parents and infants.

## Figures and Tables

**Table 1:** The participants' characteristics according to demographic and social features (*n* = 110).

Variables		Frequency (%)
*Infant-related variables*		
Gender	Female	47 (42.7)
	Male	63 (57.3)

Birth rank	First	73 (66.4)
	Second	27 (24.5)
	Third or more	10 (9.1)

Type of birth	Singleton birth	84 (76.4)
	Multiple birth	26 (23.6)

		Mean (SD)
Weight at birth (grams)		1638.7 (51.2)
Gestational birth age (weeks)		32.3 (2.6)
Length of hospitalization (days)		25.7 (19.1)

*Infant family-related variables*		Frequency (%)
Mother's education level	Illiterate	3 (2.7)
	Pre–high school diploma	29 (26.4)
	Diploma	55 (50)
	Academic	23 (20.9)

Father's education level	Illiterate	2 (1.8)
	Pre–high school diploma	38 (34.5)
	Diploma	47 (42.7)
	Academic	23 (20.9)

Mother's job	Housekeeper	91 (82.7)
	Employee	19 (17.3)

Father's job	Employee	18 (16.4)
	Self-employed	91 (82.7)
	Unemployed	1 (0.9)

Maternal delivery type	Natural	34 (30.9)
	Cesarean	76 (69.1)

		Mean (SD)
Mother's age		30.1 (6.2)
Father's age		33.6 (6.8)

**Table 2:** The amount of mothers' perception of social support provided by nurses in different supportive domains (*n* = 110).

Nurse social support domains	Range, frequency (%)			Total mean (SD)	Mean rank	*p* value^*∗*^
	Low	Moderate	High			
Affirmational	3 (2.7)	67 (60.9)	40 (36.4)	3.75(0.85)	1.85	0.1
Concrete aid	2 (1.8)	68 (61.8)	40 (36.4)	3.75(0.79)	2.00	
Affectional	3 (2.7)	56 (50.9)	51 (46.4)	3.84(0.77)	2.15	
Total social support	3 (2.7)	64 (58.2)	43 (39.1)	3.78(0.70)		

^*∗*^Friedman test.

**Table 3:** The amount of mothers' satisfaction from neonatal care in NICU (*n* = 110).

Variable	Range			Total
	Low	Moderate	High	
Mothers' satisfaction, frequency (%)	11 (10)	68 (61.8)	31 (28.2)	110 (100)
Mothers' satisfaction, mean (SD)	3.14 (0.30)	4.47 (0.49)	5.63 (0.29)	4.65 (0.83)

**Table 4:** Correlation coefficients of the Nurses Social support and mothers' satisfaction from neonatal care in NICU.

Nurse social support domains	Mothers' satisfaction (*N* = 110)	
	*r*	*p*
Affirmational	0.44	<0.001
Concrete aid	0.41	<0.001
Affectional	0.64	<0.001
Total social support	0.60	<0.001

Spearman's–Rho rank order correlation.

## Data Availability

The data used in the study are available upon reasonable request.

## Supplementary Materials

Supplementary Materials The questionnaires used in this study are presented in the supplementary file.
