# Supplementary material for: Identification of the Range of Nursing Skills Used to Provide Social Support for Mothers of Preterm Infants in Neonatal Intensive Care
Source: Crit Care Res Pract. 2021 Jan 7;2021:6697659. doi: 10.1155/2021/6697659 (PMC7810543; doi:10.1155/2021/6697659)
Supplement: Supplementary Materials — The questionnaires used in this study are presented in the supplementary file. [file 6697659.f1.docx]

**Supplementary file 1**

**Table 1.** The Social Support Questionnaire

| **Variables** | **No need** | **No at all** | **Very little** | **Little** | **Moderate** | **A lot** | **A great deal** |
| --- | --- | --- | --- | --- | --- | --- | --- |
| **Affirmational support** |  | | | | | | |
| Infant-care instructions provided |  |  |  |  |  |  |  |
| Facilitating infant development discussed |  |  |  |  |  |  |  |
| Confidence in own abilities encouraged |  |  |  |  |  |  |  |
| Instructions for monitoring infant well-being |  |  |  |  |  |  |  |
| Information about other sources of help |  |  |  |  |  |  |  |
| Independent decision-making encouraged |  |  |  |  |  |  |  |
| Nurses had sufficient time for family |  |  |  |  |  |  |  |
| **Concrete aid** |  | | | | | | |
| Directions for infant feeding |  |  |  |  |  |  |  |
| Directions for how to hold the infant |  |  |  |  |  |  |  |
| Methods to calm a wailing child |  |  |  |  |  |  |  |
| Guidance in interpreting infant communication |  |  |  |  |  |  |  |
| Practical instructions in infant care |  |  |  |  |  |  |  |
| Guidance on sources of seeking concrete support |  |  |  |  |  |  |  |
| **Emotional support (Affectional aid)** |  | | | | | | |
| Discussion on the couple’s relationship |  |  |  |  |  |  |  |
| Attention to the family |  |  |  |  |  |  |  |
| Feeling of safety in the department |  |  |  |  |  |  |  |
| Right to initial uncertainty |  |  |  |  |  |  |  |
| Personalised care |  |  |  |  |  |  |  |
| Positive feedback |  |  |  |  |  |  |  |
| Interest toward the family |  |  |  |  |  |  |  |
| Encouragement in managing at home |  |  |  |  |  |  |  |

**Table 2.** The Neonatal Instrument of Parent Satisfaction (NIPS) Questionnaire

| **Variables** | | **All of the time** | **Most of the time** | **A fair bit of the time** | | **Some of the time** | **A little of the time** | **Very little of the time** | **None of the time** |
| --- | --- | --- | --- | --- | --- | --- | --- | --- | --- |
| 1 | How often did you find the change of medical caregivers looking after your baby difficult? |  |  | |  |  |  |  |  |
| 2 | How often did these caregivers present your baby’s condition in a way which was scary or frightening? |  |  |  | |  |  |  |  |
| 3 | How often did the caregivers fail to tell you when they were going off duty? |  |  |  | |  |  |  |  |
| 4 | How often did the caregivers fail to tell you who was going to fill in while they were off duty? |  |  |  | |  |  |  |  |
| 5 | How often did you feel confused about whom to trust? |  |  |  | |  |  |  |  |
| 6 | How often did you receive conflicting information from different medical caregivers? |  |  |  | |  |  |  |  |
| 7 | How often did you feel that your baby was lost in the shuffle of a large unit? |  |  |  | |  |  |  |  |
| 8 | How often did you have difficulty finding out who your baby’s medical caregivers were? |  |  |  | |  |  |  |  |
| 9 | How often did you find the change of medical caregivers over the weekends a problem? |  |  |  | |  |  |  |  |
| 10 | How did the caregivers fail to inform you about tests or x-ray results? |  |  |  | |  |  |  |  |
| 11 | How often did you have to ask the medical caregivers to repeat explanations several times? |  |  |  | |  |  |  |  |
| 12 | How often were you uncertain who to talk to about your baby’s condition? |  |  |  | |  |  |  |  |
| 13 | How often did the medical caregivers fail to inform you completely about the results of a procedure? |  |  |  | |  |  |  |  |
| 14 | How often did the caregivers keep you waiting for results of tests? |  |  |  | |  |  |  |  |
| 15 | How often were you informed about something after-the-fact or by accident? |  |  |  | |  |  |  |  |
|  | | **None of the time** | **Very little of the time** | **A little of the time** | | **Some of the time** | **A fair bit of the time** | **Most of the time** | **All of the time** |
| 16 | How often did you feel that you knew who was who?? |  |  |  | |  |  |  |  |
| 17 | How often did the medical caregivers volunteer how they felt about your baby’s condition? |  |  |  | |  |  |  |  |
|  |  | **Not really satisfied** | **Generally satisfied, but*** | **Generally satisfied, but**** | | **Quite satisfied** | **Mostly satisfied** | **Very satisfied** | **Completely satisfied** |
| 18 | How satisfied were you with the way the medical caregivers prepared you for your baby’s stay in the NICU? |  |  |  | |  |  |  |  |
| 19 | How satisfied were you with the extent to which the caregivers kept you informed as your baby’s condition changed? |  |  |  | |  |  |  |  |
| 20 | How satisfied were you with how often the caregivers offered to meet with you in private? |  |  |  | |  |  |  |  |
| 21 | How satisfied were you with the number of meetings arranged with your baby’s doctors to discuss what you might expect for your baby in the future? |  |  |  | |  |  |  |  |
| 22 | How satisfied were you with how the medical caregivers told you about the long-term expectation or outlook for your child? |  |  |  | |  |  |  |  |
| 23 | How satisfied were you with how much the caregivers were sensitive to the other pressures in your life? |  |  |  | |  |  |  |  |
| 24 | How satisfied were you with the extent to which the caregivers offered personal opinions or experiences about your baby’s future condition? |  |  |  | |  |  |  |  |
| * Significant areas of dissatisfaction  ** Minor areas of dissatisfaction | | | | | | | | | |
